# Research and discussion on the evaluation scheme of reagent lot‐to‐lot differences in 16 chemiluminescence analytes, established by the EP26‐A guidelines of the CLSI

**DOI:** 10.1002/jcla.23675

**Published:** 2020-12-03

**Authors:** Ran Tao, Chenli Zhang, Min Liu, Miao Yang, Wenying Gao, Jianbo Chen, Nanxun Mo, Yating Cheng, Jun He, Qin Xie

**Affiliations:** ^1^ Laboratory Diagnosis Department Guangzhou Kingmed Center for Clinical Laboratory Guangzhou China; ^2^ Clinical laboratory medicine Guangzhou Medical University Guangzhou China; ^3^ Laboratory Diagnosis Department Taiyuan Kingmed Center for Clinical Laboratory Taiyuan China; ^4^ Laboratory Diagnosis Department Changsha Kingmed Center for Clinical Laboratory Changsha China

**Keywords:** chemiluminescence analyte, CLSI EP26‐A, lot verification

## Abstract

**Background:**

Verification of new reagent lots is a part of the crucial tasks in clinical laboratories. The Clinical and Laboratory Standards Institute (CLSI) EP26‐A guideline provides laboratories with an evaluation method for reagent verification. The purpose of this study was to compare the performance of EP26‐A with our laboratory reagent lot verification protocol and get the final scheme.

**Method:**

16 chemiluminescence analytes including estradiol (E2), progesterone (P), ferritin (FER), cortisol (COR),carbohydrate antigen 153 (CA153), and free prostate‐specific antigen (FPSA). were prospectively evaluated in two reagent lots. The laboratory's lot verification process included evaluating 5 patient samples with the current and new lots and acceptability according to a predefined criteria. For EP26‐A, method imprecision data and critical differences at medical decision points were important factors affecting the sample size requirements and rejection limits.

**Result:**

The number of samples required for EP26‐A was 3 to 12, of which P, CA153, and FPSA had increased by more than 5 samples compared with the current protocol. Of the 16 chemiluminescence analytes, 11 had higher rejection limits when using EP26‐A than the current laboratory scheme. Our current protocol and EP26‐A were in agreement in 32 of the 32 (100%) paired verifications.

**Conclusion:**

The EP26‐A protocol is an important tool to find the differences between reagent lots, and it makes up for the loopholes in the statistical efficiency, sample concentration and quantity, and the selection of rejection limits in the current protocol.

## Graphical abstract

From studies published in China, the scope of EP26‐A application is far lower than that of other guidelines. The main advantages of the EP‐26A scheme are as follows: 1. assess target concentration of analyte; 2. combined use of analytical compounds precision performance; and 3. statistical efficacy was proposed for 0.8. The EP26‐A protocol is an important tool to find the differences between reagent lots, and it makes up for the loopholes in the statistical efficiency, sample concentration and quantity, and the selection of rejection limits in the current protocol.

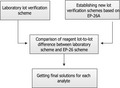

## 1 INTRODUCTION

In recent years, with the great progress in technology, medical laboratories play an increasingly important role in clinical diagnosis and treatment. Doctors use laboratory test results to assist them in diagnosis, guide treatment, and monitor prognosis. The precision and accuracy of test results and the performance consistency of different reagent lots are the prerequisites to ensure the effectiveness of the results. Performance evaluation of different reagent lots is an indispensable step to ensure consistency of test results.^1^ Improper evaluation methods or inability to detect reagents that are disqualified due to defects in the raw materials, or as a result of issues with the transportation and storage, are bound to have a significant impact on clinical diagnosis and treatment. The “Medical Laboratory Quality and Capability Accreditation Guidelines” 5.3.2.3 of the China National Accreditation Committee for Conformity Assessment (CNAS‐CL02:2012) requires evaluation of each new lot before putting it into clinical use. Laboratories need to compare the new and currently used lots.^2^

In 2013, the American Clinical and Laboratory Standards Institute (CLSI) issued the EP26‐A guidelines, providing lot evaluation methods for laboratories.^3^ From studies published in China, the scope of EP26‐A application is far lower than that of other guidelines. Most laboratories implement performance evaluation following the A.5 requirements. However, chemiluminescence detection reagents exist large differences between lots due to the affinity and purity of the antibodies, and the maximum allowable error variations between laboratories. Even if a laboratory indicates that a lot is abnormal, the manufacturer lacks the motivation to do anything about it unless it receives feedback from other users as well. A set of scientific and professional lot verification schemes is of great necessity. In 2017, Brooke M. Katzman compared the evaluation effectiveness of EP26‐A, using 20‐sample verification schemes.^4^ Because our program is quite different from that of the Mayo Clinic, we developed plans for 16 chemiluminescence analytes, based on the EP26‐A guidelines, and obtained an implementation plan by reviewing past data.

## 2 MATERIALS AND METHODS

### 2.1 Samples and analytes

All lot verification samples were used on actual patients' samples. Analytes evaluated included: estradiol (E2), progesterone (P), testosterone (T), follicle‐stimulating hormone (FSH), ferritin (FER), vitamin B12 (VB12), chorionic gonadotropin (hCG), C‐peptide (C‐P), cortisol (COR), alpha‐fetoprotein (AFP), carcinoembryonic antigen (CEA), carbohydrate antigen 125 (CA125), carbohydrate antigen 153 (CA153), carbohydrate antigen 199 (CA199), total prostate‐specific antigen (TPSA), and free prostate‐specific antigen (FPSA). Among them, E2, P, T, FSH, FER, and VB12 were detected by the Abbott I2000 chemiluminescence instrument, and the other analytes were detected by Roche Combas 8000 c601.^5^, ^6^ The reagents were all matching reagents from various manufacturers.

### 2.2 Reagent lot verifications

#### 2.2.1 Laboratory scheme

Samples from five patients in a stable period were selected for all analytes. The concentration covered the measured range of analysis, and the old and new lots were used for detection. Bias was calculated based on the following formula:

Bias (%)=(oldlotresult‐newlotresult)×100/oldlotresult

1/3 of the total allowable error (TEA) of each analyte, as specified in the “Quality Evaluation Standard of Interventricular Quality in Clinical Examination,” issued by the China Health Commission, was taken as the judgment standard.^7^ If the bias was less than or equal to the standard in at least four of the five samples, then the new lot reagent could be used. Otherwise, it was not acceptable. This evaluation method will be referred to hereinafter as the old scheme.

#### 2.2.2 Lot verification based on the EP26‐A guidelines

The critical difference, CD, was determined based on Appendix D1 of the EP26‐A guidelines. To reduce a class of errors, the Z‐value was assigned a unilateral value of a 99% confidence interval (3.09). Based on this, CD = 3.09 × 1.41 × CV_WRL_ = 4.36 × CV_WRL_. Imprecision data came from performance verification. If the within‐reagent lot imprecision and repeatability at the same medical decision level could not be obtained, the interpolation method was used. If the medical decision level was within the laboratory‐evaluated concentration range, the imprecision of the medical decision level was estimated by the TREND and OFFSET functions. If the target concentration was not within the concentration range already evaluated in the laboratory, an imprecision close to the concentration was used for the evaluation. The efficacy of the statistical analytes was 0.80. This evaluation method will be referred to hereinafter as the new scheme.

**FIGURE 1:**
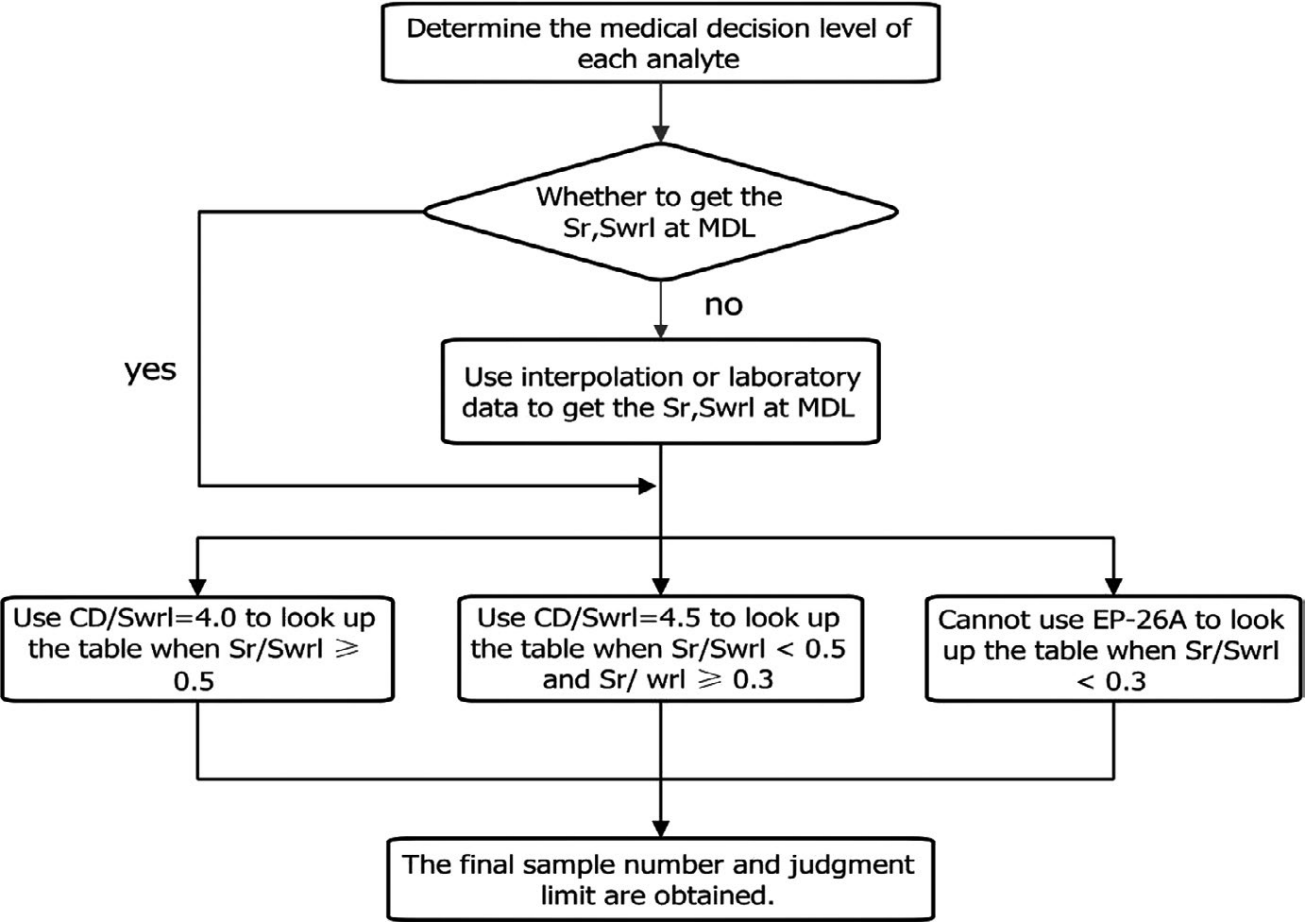
Evaluation steps of the improved EP26‐A protocol. MDL, medical decision level; Sr, repeatability (within‐run imprecision); Swrl, within‐reagent lot imprecision; CD, critical differences

The sample number and judgment scope of each analyte were obtained following the steps detailed in Figure 1. In the actual evaluation, the samples selected according to the evaluation results were tested with new and old lots. The bias was calculated based on the formula mentioned above. The average bias was obtained at the medical decision level, and a decision was then made as to whether the average bias is less than the judgment limit. The judgment limit was obtained by looking up in the table. If the average bias was less than the judgment limit, the new lot was acceptable. Otherwise, it was unacceptable.

### 2.3 Comparison of scheme effect

The two methods were compared for a sample number range of judgment limits, judgment results of previous data using the new scheme, and the percentage of the previous evaluation bias from the judgment limit.

### 2.4 Scheme decision

Deciding on the final scheme according to the clinical use of each analyte, the number of evaluation samples, and the judgment limit. If there was no result in the look‐up table, the current method was retained; if the evaluation sample size was more than 30 in the look‐up table analyte, the current method was retained. If the analyte was used for disease diagnosis, we chose the medical decision level on the concentration. Otherwise, we retained the current linear range. In terms of judgment limit, if the ratio of the new scheme to the old one was between 0.5 and 2, we chose the new scheme. Beyond this range, we followed the analysis results.

## 3 RESULT

### 3.1 Results of the newly proposed evaluation method

Table 1 shows the results of the evaluation based on the new scheme. Sixteen of the 32 concentrations (50%) were interpolated to obtain the imprecision of the target concentration. The Sr/Swr of all analytes was found to be greater than 0.3, using a judgment limit of 0.7 CD, and the required quantity samples ranged between 3‐12 per test. Among them, a sample size of 10 analytes was less than or equal to five, and the number of samples of six analytes was greater than five. CA153 and P had much larger sample size than other analytes, with 11 and 12 samples, respectively.

**TABLE 1:** Result of the new scheme's evaluation

Analyte	MDL	Swrl	Sr	Sr/Swrl	CD	Judgment limit	Number of patient samples	Total number of samples
E2 (pg/ml)	98^a^	3.49	2.67	0.77	15.22	0.7CD	2	4
	398^a^	3.09	2.58	0.83	13.47	0.7CD	2	
P (ng/ml)	0.5	5.98	4.09	0.68	26.07	0.7CD	3	12
	29.8	3.32	1.83	0.55	14.48	0.7CD	9	
T (nmol/L)	7.28^a^	4.38	4.06	0.93	19.1	0.7CD	2	4
	28^a^	4.46	4.13	0.93	19.45	0.7CD	2	
FSH (mIU/ml)	9.6^a^	3.05	3.58	1.17	13.3	0.7CD	2	4
	46^a^	3.37	3.47	1.03	14.69	0.7CD	2	
FER (ng/ml)	63^a^	4.69	2.83	0.60	20.45	0.7CD	3	6
	580^a^	4.66	3.21	0.69	20.32	0.7CD	3	
VB12 (pg/ml)	387^a^	5.14	4.54	0.88	22.41	0.7CD	2	4
	992^a^	5.62	4.1	0.73	24.5	0.7CD	2	
HCG (mIU/ml)	4.7^a^	3.64	4.85	1.33	15.87	0.7CD	2	4
	172^a^	3.64	4.85	1.33	15.87	0.7CD	2	
C‐P (ng/ml)	1.7^a^	2.44	0.89	0.36	11.03^b^	0.7CD	2	3
	9.4^a^	2.41	1.02	0.42	10.80^b^	0.7CD	1	
COR (nmol/L)	105^a^	4.66	4.73	1.02	20.32	0.7CD	2	4
	810^a^	3.48	3.06	0.88	15.17	0.7CD	2	
AFP (IU/ml)	8.44	4.02	2.72	0.68	17.53	0.7CD	3	6
	200.2	2.88	1.92	0.67	12.56	0.7CD	3	
CEA (ng/ml)	3.82	3.61	2.44	0.68	15.74	0.7CD	3	6
	71.83	3.41	2.05	0.60	14.87	0.7CD	3	
CA125 (U/ml)	31.96	2.74	2.15	0.78	11.95	0.7CD	2	4
	209.89	2.23	1.55	0.70	9.72	0.7CD	2	
CA153 (U/ml)	24.65	3.46	1.7	0.49	15.57^b^	0.7CD	2	11
	111.79	3.16	1.81	0.57	12.64^b^	0.7CD	9	
CA199 (U/ml)	30.5	3.06	2.46	0.80	13.34	0.7CD	2	4
	199.3	2.35	2	0.85	10.25	0.7CD	2	
TPSA (ng/ml)	0.11	3.48	2.6	0.75	15.17	0.7CD	2	5
	15.69	2.22	1.5	0.68	9.68	0.7CD	3	
FPSA (ng/ml)	0.066	4.6	1.6	0.35	23.00^b^	0.7CD	1	10
	13.07	2.72	1.35	0.50	11.86	0.7CD	9	

^a^ MDL, Interpolation was used to estimate the imprecision of medical decision level (MDL).

^b^ CD, Since the table cannot be used when Sr/Swrl < 0.5 or CD/Sr = 4.36, a value of CD/Sr = 4.5 was used to look up the table.

### 3.2 Comparisons of the two schemes

Table 2 shows that the number of evaluations of six of the analytes in the new scheme was higher than in the old scheme. The items with the largest increase were P and CA153. The new scheme was wider than the old one in 11 analytes. The judgment limit was narrower in the new scheme than that of the old scheme in four analytes, and the judgment range of the two schemes for FPSA was the same. In two analytes (P and B12), the difference in the judgment limit of the two schemes was more than 2.

**TABLE 2:** Comparisons between the two schemes

Analyte	Number of samples for the new scheme	Number of changes	MDL	Judgment limit of the new scheme	Judgment limit of the old scheme	Ratio of new to old scheme
E2 (pg/ml)	4	−1	98	±10.44	±8.09	1.29
			398	±37.53	±32.84	1.14
P (ng/ml)	12	+7	0.47	±0.09	±0.04	2.25
			29.8	±3.02	±2.46	1.22
T (nmol/L)	4	−1	7.28	±0.97	±0.60	1.62
			28	±3.81	±2.31	1.65
FSH (mIU/ml)	4	−1	9.6	±0.89	±0.79	1.13
			46	±4.73	±3.80	1.24
FER (ng/ml)	6	+1	63	±9.02	±5.20	1.73
			580	±82.49	±47.85	1.72
VB12 (pg/ml)	4	−1	387	±60.71	±31.93	1.9
			992	±170.15	±81.84	2.07
HCG (mIU/ml)	4	−1	4.7	±0.52	±0.39	1.33
			172	±19.11	±14.19	1.35
C‐P (ng/ml)	3	−2	1.7	±0.13	±0.14	0.92
			9.4	±0.81	±0.78	1.03
COR (nmol/L)	4	−1	105	±14.93	±8.66	1.72
			810	±106.62	±66.83	1.60
AFP (IU/ml)	6	+1	8.44	±1.04	±0.70	1.48
			200.2	±17.60	±16.52	1.07
CEA (ng/ml)	6	+1	3.82	±0.42	±0.32	1.31
			71.83	±7.48	±5.93	1.26
CA125 (U/ml)	4	−1	31.96	±2.67	±2.64	1.01
			209.89	±14.29	±17.32	0.83
CA153 (U/ml)	11	+6	24.65	±2.69	±2.03	1.33
			111.79	±9.89	±9.22	1.07
CA199 (U/ml)	4	−1	30.54	±2.85	±2.52	1.13
			199.3	±14.29	±16.44	0.87
TPSA (ng/ml)	5	0	0.11	±0.01	±0.01	1
			15.69	±1.06	±1.29	0.82
FPSA (ng/ml)	10	+5	0.066	±0.01	±0.01	1
			13.07	±1.08	±1.08	1

### 3.3 Using the new scheme to evaluate historical data

In 2019, a total of 49 lots, covering the 16 analytes, involved 245 tests. Only 62 tests, covering 14 analytes, were at the medically determined level, accounting for 25.3% of the total number of tests. Sample results of the previous medical decision level were judged by the new scheme (Table 3). All 32 paired tests (100%) were consistent and passed the evaluation.

**TABLE 3:** Using the new scheme to judge the historical data results

Analyte	MDL	Number of evaluation samples per batch	Evaluation variance	Judgment limit of new scheme	Percentage	Evaluation result
E2 (pg/ml)	98	1	1.00	10.44	9.58%	Pass
		1	6.00	10.44	57.47%	Pass
		1	5.00	10.44	47.89%	Pass
	398	1	−5.00	37.53	−13.32%	Pass
		1	2.00	37.53	5.33%	Pass
		1	14.00	37.53	37.30%	Pass
P (ng/ml)	29.8	1	1.90	3.02	62.92%	Pass
		1	−1.33	3.02	−44.05%	Pass
		1	0.74	3.02	24.51%	Pass
		2	−0.89	3.02	−29.31%	Pass
		1	0.57	3.02	18.88%	Pass
T (nmol/L)	7.28	1	0.34	0.97	35.05%	Pass
		1	0.03	0.97	3.09%	Pass
		1	0.53	0.97	54.64%	Pass
	28	1	−0.43	3.81	−11.29%	Pass
		1	0.73	3.81	19.16%	Pass
		1	1.35	3.81	35.43%	Pass
FSH (mIU/ml)	9.6	1	0.07	0.89	7.87%	Pass
	46	1	−0.69	4.73	−14.59%	Pass
		1	−0.26	4.73	−5.50%	Pass
FER (ng/ml)	63	1	4.47	9.02	49.57%	Pass
		1	0.95	9.02	10.51%	Pass
	580	1	−46.77	82.49	−56.70%	Pass
VB12 (pg/ml)	387	1	7.00	60.71	11.53%	Pass
		1	10.00	60.71	16.47%	Pass
		1	−1.00	60.71	−1.65%	Pass
	992	1	−10.00	170.15	−5.88%	Pass
		1	−25.00	170.15	−14.69%	Pass
HCG (mIU/ml)	4.7	1	−0.27	0.52	−51.71%	Pass
	172	1	0.10	19.11	0.52%	Pass
		1	0.20	19.11	1.05%	Pass
C‐P (ng/ml)	1.7	1	−0.03	0.13	−23.0%	Pass
		2	0.04	0.13	30.7%	Pass
	9.4	1	0.01	0.81	1.24%	Pass
		1	0.01	0.81	1.24%	Pass
COR (nmol/L)	105	1	0.70	2.46	28.40%	Pass
		2	−2.10	2.46	−85.20%	Pass
	810	1	10.30	106.62	9.66%	Pass
		2	−18.40	106.62	−17.26%	Pass
AFP (IU/ml)	8.44	1	0.21	1.04	20.28%	Pass
		1	0.11	1.04	10.62%	Pass
		2	−0.46	1.04	−44.42%	Pass
	200.2	1	4.90	17.60	27.85%	Pass
		1	−3.10	17.60	−17.62%	Pass
		1	−9.50	17.60	−53.99%	Pass
CEA (ng/ml)	3.82	1	0.21	0.42	49.90%	Pass
		2	−0.03	0.42	−7.13%	Pass
	71.83	1	4.48	7.48	59.93%	Pass
		3	−0.91	7.48	−12.17%	Pass
CA125 (U/ml)	31.96	1	−0.30	2.67	−11.22%	Pass
CA153 (U/ml)	24.65	1	1.60	2.69	59.55%	Pass
		1	0.09	2.69	3.35%	Pass
	111.79	1	7.30	9.89	73.80%	Pass
CA199 (U/ml)	30.54	1	0.02	2.85	0.70%	Pass

### 3.4 Getting the final scheme according to the actual situation

The final proposal is shown in Table 4.

**TABLE 4:** Final assessment results

Analyte	Clinical efficacy	MDL	Sample retention	Result
E2 (pg/ml)	Auxiliary diagnosis	98	Keep samples at any time	EP‐26
		398	Keep the sample in advance	
P (ng/ml)	Auxiliary diagnosis	0.47	Keep samples at any time	EP‐26
		29.8	Keep samples at any time	
T (nmol/L)	Auxiliary diagnosis	7.28	Keep samples at any time	EP‐26
		28	Keep the sample in advance	
FSH (mIU/ml)	Auxiliary diagnosis	9.6	Keep samples at any time	EP‐26
		46	Keep samples at any time	
FER (ng/ml)	Auxiliary diagnosis	63	Keep samples at any time	EP‐26
		580	Keep the sample in advance	
VB12 (pg/ml)	Auxiliary diagnosis	387	Keep samples at any time	EP‐26
		992	Keep samples at any time	
HCG (mIU/ml)	Diagnosis/monitoring	4.7	Keep the sample in advance	EP‐26
		172	Keep the sample in advance	
C‐P (ng/ml)	Auxiliary diagnosis	1.7	Keep the sample in advance	EP‐26
		9.4	Keep the sample in advance	
COR (nmol/L)	Auxiliary diagnosis	105	Keep samples at any time	EP‐26
		810	Keep the sample in advance	
AFP (IU/ml)	Diagnosis/monitoring	8.44	Keep samples at any time	EP‐26
		200.2	Keep the sample in advance	
CEA (ng/ml)	Prognosis monitoring	3.82	Keep samples at any time	EP‐26
		71.83	Keep the sample in advance	
CA125 (U/ml)	Prognosis monitoring	31.96	Keep samples at any time	Both methods can be used
		209.89	Keep the sample in advance	
CA153 (U/ml)	Prognosis monitoring	24.65	Keep samples at any time.	EP‐26
		111.79	Keep the sample in advance	
CA199 (U/ml)	Prognosis monitoring	30.54	Keep the sample in advance	Both methods can be used.
		199.3	Keep the sample in advance	
TPSA (ng/ml)	Auxiliary diagnosis	0.11	Keep samples at any time	EP‐26
		15.69	Keep the sample in advance	
FPSA (ng/ml)	Auxiliary diagnosis	0.066	Keep samples at any time	EP‐26
		13.07	Keep the sample in advance	

## 4 DISCUSSION

Owing to the changes in raw materials through the production process, and the decline in activity during transportation and storage, clinical laboratories should verify the performance of any new lot before using it for the detection of clinical samples. Between‐reagent lot variation can affect results for QC materials, patient samples, or both. It is possible that a difference in patient sample results occurs between two different reagent lots, but there is no difference seen for QC results. This is because the manufacturing process for QC materials has a significant impact on the matrix of these samples and the reagent manufacturer's first concerns must be accuracy and consistency with patient sample results. In addition, QC material supplied with the reagents may be “optimized” to perform correctly with each new reagent lot. Therefore, it is important that reagent lot‐to‐lot evaluations be performed using patient samples for all reagent lot changes. Ideally, the same evaluation scheme would be used by multiple users of the same reagent factory. Problems with abnormal lot can then be found,^8^ confirmed, and the laboratories and manufacturers could be urged to investigate the issue together. Not all unqualified reagents could be detected if the design of the evaluation scheme is unscientific, relying on statistical efficiency, sample concentration and number, and different selection of key errors. The EP26‐A protocol makes up for the above loopholes. In the study by Brooke M. Katzman, although the new scheme was not used in the end, the self‐designed method was further improved through the comparison between the two methods.

In the process of implementing the new scheme, there are three points worth learning: 1. The new scheme emphasizes that the selection of samples should be near the medical decision level and that analytes of auxiliary diagnosis should especially consider the concentration. Of the 245 tests recorded in our historical evaluation, only 62 tests (25.3%) were near the medical decision level; 2. The calculated result bias of the new scheme uses the total bias of each medical decision level, and the positive and negative biases are considered to be random errors. The additive elimination, and the fact that the bias of each sample was calculated separately by the old scheme, might lead to false‐positive results. The above ideas suggest that evaluation of laboratory data should be based on clinical services and consider the key concentration; 3. The new scheme requires that the concentration of within‐run imprecision in the laboratory would be consistent with that of the within‐reagent lot imprecision, and near the medical decision level. In the past, there was a difference in the evaluated concentration of within‐run imprecision and within‐reagent lot imprecision of some analytes in our laboratory. In this article, 50% of the concentrations were estimated by the interpolation method.

From the results of the evaluation, the number of samples of FPSA, CA153, and P was higher by more than five each. The judgment limit of more than 90% of the concentrations in the two methods was between 1 and 2. These findings indicate that the allowable limit of the new scheme is generally wider than that of the old scheme. Since the difference in the limit of judgment for P and VB12 was greater than 2, we analyze the reasons from the following perspectives: 1. Swrl was too large. The calculation of inter‐batch imprecision was mixed with reagent inter‐batch differences: in the past, the statistical time of inter‐batch imprecision in our laboratory was limited by the replacement of quality control lots. The problem is that inter‐batch differences lead to an increase in inter‐batch imprecision variation. The bias caused by the inter‐batch differences should be considered as the test noise, and thus the smaller the better. The appropriate statistical interval of inter‐batch precision shall be subject to any replacement of the quality control lot and the reagent lot. Reviewing the history of quality control, The CV of P low concentration reagent lots was 5.82% and 6.02%, respectively, and the total CV was 5.98%. The CV of each lot of high concentration VB12 was 5.02%, 4.35%, and 3.27%, the total CV was 5.62%, and the respective total CV was less than 1/3 of the maximum allowable error. We compared the inter‐batch precision of other laboratories in Jinyu. P range was between 2.48% and 7.52%, and at our laboratory it was 66%. Range of VB12 was: 3.1%, Murray 6.28%, and at our laboratory it was 58%. Therefore, we need to find out the reasons for the decrease in Swrl. 2. Whether the maximum allowable error of this concentration was appropriate. Referring to the external quality evaluation standard of ESfEQA GmbH in Germany, the judgment limit of P in 0.47 ng/ml is 0.3 ng/ml, and the limit of the judgment of VB12 in 992 pg/ml is 89 pg/ml. In our laboratory, we use the percentage form the maximum allowable error for P, and the low concentration is narrow. Finally, we tried to evaluate the above two analytes, using the method proposed by George Klee to calculate the allowed difference based on patient historical data. Based on this method, the allowed low concentration difference of P was 0.06 ng/ml, which was between the CD of the two regimens. The high allowed difference of VB12 was 152.25 pg/ml, which was closer to the EP26‐A result. To sum up, we chose to use the EP26‐A method for subsequent verification.

Using the new scheme to evaluate the historical data, although all the previous data passed, only 25.3% of the tests were at the medical decision level. The sample number required by FER, AFP, CEA, FPSA, CA153, and P was greater than 5, and the significant lot‐to‐lot difference could not be found due to the small amount of previous data.

In the choice of final adoption scheme, as the clinical use of CEA, CA125, CA153, and CA199 is mainly for monitoring function, the judgment limit of CEA and CA153 was greater than 1, and the ratio of previous evaluation bias to judgment limit was more than 50%, so choose a new scheme. The ratio of past bias to the judgment limit of CA125 and CA199 was‐11.22% and 0.7%, respectively, so both schemes are acceptable.

In this article, the EP26‐A method was used to evaluate the applicability of 16 chemiluminescence analytes in our laboratory. We think that the new scheme is very helpful for the quality improvement of the laboratory. The limitation of this article lies in that there are two aspects of the new scheme that should still be clarified: 1. Whether the CD in this article is equal to the maximum allowable error. For this article, we chose the method detailed in appendix D1, which is more robust. This appendix is completely based on laboratory detection performance, and does not depend on the unrelated opinion and performance of other laboratories (EQA). 2. Part of this article is about calibration, which is a mandatory requirement before evaluating the differences. This calibration is quite different from the old scheme and increases the complexity and cost of laboratory evaluation.

In conclusion, reasonable and scientific evaluation is important and urgent, and the introduction of the EP26‐A scheme is an important tool to find the differences between reagent lots, The implementation of the scheme can promote the applicability of laboratory review quality indicators and benefit the quality risk education of personnel.

## CONFLICT OF INTEREST

The authors declare that there are no competing interests associated with the manuscript.

## Data Availability

The data that support the findings of this study are available from the corresponding author upon reasonable request.
